# Angina Pectoris

**Published:** 1829

**Authors:** 


36. *Angina Pectoris. Nervous System.*——Having long thought the nervous system primarily affected in all diseases not arising from mechanical violence, we are too much pleased with the following remarks of the Editors of the Medico-

Chirurgical Review, (January, 1829) not to recommend them to the perusal of our readers; especially the younger class, whose pathological views have not yet become immoveably anchored by a weight of prejudice and error. The writer after giving an account of a case of Angina pectoris reported in Dr. Farre's semi-annual journal, concludes with these remarks:

That the disease termed angina pectoris may depend on more than one pathological condition of the heart cannot now be doubted; but, that it is, in the great majority of instances, a neuralgic affection of the organ, we firmly believe. In most of the cases which we have had an opportunity of examining, the muscular structure was flabby and wasted—or loaded with fat. What do we see in neuralgic affections elsewhere? Wasting and weakness of the muscles. Death, in an angina pectoris, we believe to result from one of two conditions—spasm, or momentary paralysis of the organ. From spasm, when the patient dies in agonies, as is sometimes the case—from momentary paralysis, when he dies instantaneously, without suffering. In the latter case, it is of *syncope*, in fact, which the individual expires—and the disease, indeed, has been denominated "syncope anginosa." Why should we wonder that the heart may be affected with temporary paralysis, as well as other muscles? There is scarcely a muscle in the body, voluntary or involuntary, that has not been found to be the seat of temporary paralysis.—This temporary loss of power in the heart, must of course, be fatal, if it last beyond a certain time. In common syncope, from emotions of the mind, loss of blood, or other causes, power is restored before the vital spark is fled—though not always, for death sometimes takes place, even from this momentary cessation of action in the central organ of the circulation. It is hardly necessary to say, that to attribute the disease, in the case above narrated, to the specks of ossification on the coronary arteries, when other and far more serious lesions were discovered, (though perhaps still more lay behind) on dissection, is a sad specimen of philosophising in medicine! We would recommend to those young aspirants for professional fame, who are, as yet, unencumbered with the harrassing duties of private practice, to direct their attention to the pathology of the visceral nerves. We cannot indeed, expect to discover the causes of functional disorder in the nervous system so readily as in the arterial system; but still we believe there is much appreciable lesion to be detected by accurate dissection. In the mean time, we cannot but deplore the rage for tracing disorders of the human machine entirely to physical alterations of structure, knowing as we do, that all diseases, or almost all, originate in disordered function, connected with, if not entirely dependent on the nervous system. According, indeed, to some modern medical philosophers, the brain and nerves are almost useless parts of the animal machine—while the mechanical or physical agencies are every thing. The laws of hydraulics and chemistry are soon to supersede the laws of vitality—and the *morale* is soon to be absorbed in the *physique*.

